# Supplementary material for: Splice-Junction-Based Mapping of Alternative Isoforms in the Human Proteome
Source: Cell Rep. Author manuscript; Available in PMC 2020 Jan 15. (PMC6961840; doi:10.1016/j.celrep.2019.11.026)
Supplement: 2 [file NIHMS1546469-supplement-2.zip › DF1/PXD006675/PulmonaryVein/PulmonaryVein_15_TTN_VEAEPAEVTEFR.pdf]

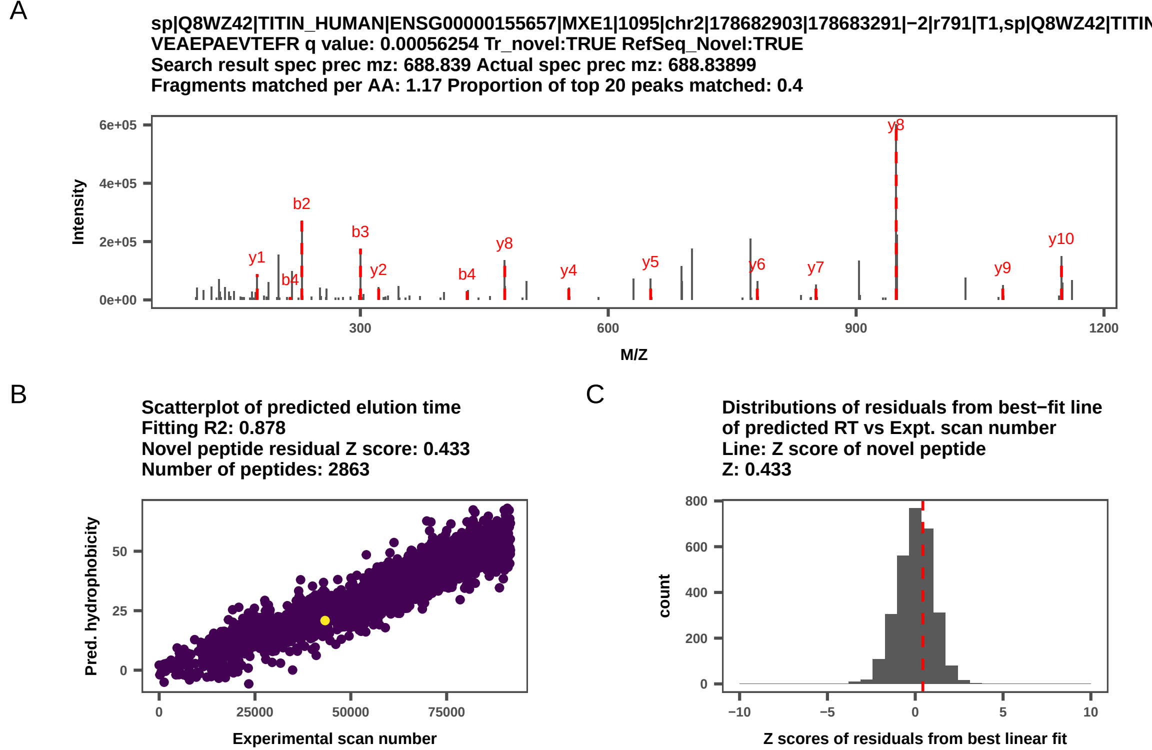
A
sp|Q8WZ42|TITIN_HUMAN|ENSG00000155657|MXE1|1095|chr2|178682903|178683291|−2|r791|T1,sp|Q8WZ42|TITIN
VEAEPAEVTEFR q value: 0.00056254 Tr_novel:TRUE RefSeq_Novel:TRUE
Search result spec prec mz: 688.839 Actual spec prec mz: 688.83899
Fragments matched per AA: 1.17 Proportion of top 20 peaks matched: 0.4
Intensity
6e+05
4e+05
2e+05
0e+00
y1
b4
b2
b3
y2
b4
y8
y4
y5
y6
y7
y8
y9
y10
300
600
900
1200
M/Z
B
Scatterplot of predicted elution time
Fitting R2: 0.878
Novel peptide residual Z score: 0.433
Number of peptides: 2863
Pred. hydrophobicity
50
25
0
0
25000
50000
75000
Experimental scan number
C
Distributions of residuals from best−fit line
of predicted RT vs Expt. scan number
Line: Z score of novel peptide
Z: 0.433
count
800
600
400
200
0
−10
−5
0
5
10
Z scores of residuals from best linear fit
